# Eradication of *Porphyromonas gingivalis* Persisters Through Colloidal Bismuth Subcitrate Synergistically Combined With Metronidazole

**DOI:** 10.3389/fmicb.2021.748121

**Published:** 2021-10-20

**Authors:** Chuan Wang, Xuan Li, Tianfan Cheng, Hongzhe Sun, Lijian Jin

**Affiliations:** ^1^Division of Periodontology and Implant Dentistry, Faculty of Dentistry, The University of Hong Kong, Hong Kong SAR, China; ^2^Department of Chemistry, The University of Hong Kong, Hong Kong SAR, China

**Keywords:** *Porphyromonas gingivalis*, persisters, eradication, bismuth drugs, metronidazole

## Abstract

Microbial persisters enable the development of certain intrinsic strategies for survival with extreme tolerance to multiple antimicrobials. *Porphyromonas gingivalis* is considered to be the “keystone” periodontopathogen. Indeed, periodontitis, as a highly common inflammatory disease, is the major cause of severe tooth loss and edentulism in adults globally, and yet it is crucially involved in various systemic comorbidities like diabetes. We have recently revealed *P. gingivalis* persisters-induced perturbation of immuno-inflammatory responses and effective suppression of this key pathogen by bismuth drugs. This study further explored novel approaches to eradicating *P. gingivalis* persisters through synergistic combination of colloidal bismuth subcitrate (CBS) with traditional antibiotics. *P. gingivalis* (ATCC 33277) cells in planktonic and biofilm states were cultured to stationary phase, and then treated with metronidazole (100 mg/L), amoxicillin (100 mg/L), CBS, (100 μM) and combinations of these medications, respectively. Persister survival rate was calculated by colony-forming unit. Cell viability and cytotoxicity of CBS were assessed in human gingival epithelial cells (HGECs). Notably, CBS combined with metronidazole enabled the effective eradication of *P. gingivalis* persisters in planktonic mode, and nearly eliminated their existence in biofilm mode. Importantly, CBS exhibited no effects on the viability of HGECs, along with minimal cytotoxicity (<5%) even at a high concentration (400 μM). This pioneering study shows that *P. gingivalis* persisters could be well eliminated via the synergistic combination of CBS with metronidazole. Our findings may contribute to developing novel approaches to tackling periodontitis and inflammatory systemic comorbidities.

## Introduction

Microbial persisters as a tiny subset of microorganisms can enter or be triggered to become a “dormant” and “non-dividing” state, with high tolerance to multiple antimicrobials. These persisters normally take up less than 0.1% of the whole population without heritable genetic mutations, as the descendants of persisters are still sensitive to antimicrobial treatment and generate a similar proportion of persisters (Lewis, 2010; Balaban et al., 2019). As persisters can survive under antimicrobials treatment and resume growth after the cessation of treatment, these noxious cells have been claimed to critically account for the relapse and/or recalcitrance of common infectious/inflammatory diseases in humans (Lewis, 2010; Fauvart et al., 2011).

Periodontitis, as a serious inflammatory disease, is one of the major global oral disease burdens (Pihlstrom et al., 2005; Jin et al., 2011, 2016; Kassebaum et al., 2014; Tonetti et al., 2017). *Porphyromonas gingivalis* is considered to be the “keystone” periodontopathogen (Hajishengallis et al., 2012) and it critically contributes to the shift of host-microbe symbiosis to dysbiosis even at a low abundance, leading to dysregulated immuno-inflammatory responses and periodontal destruction (Hajishengallis et al., 2011; Honda, 2011). Notably, periodontitis is closely linked with various systemic diseases and disorders, so called inflammatory comorbidities, and indeed *P. gingivalis* plays essential roles in the etiopathogenesis of these diseases (Hajishengallis, 2015; Hajishengallis and Chavakis, 2021). Recently, our group reported for the first time the profile of *P. gingivalis* persisters and their underlying survival mechanisms (Li et al., 2018), and surprisingly metronidazole-treated *P. gingivalis* persisters maintain their virulence factors, such as the ability to adhere to and invade human gingival epithelial cells (HGECs), and perturb immuno-inflammatory responses (Wang et al., 2020). These findings may to some extent account for the hardship to effectively control periodontitis and prevent its recurrence, especially in susceptible individuals. Herein, the working hypothesis is that targeting *P. gingivalis* persisters might be a critical strategy to tackle periodontitis and other *P. gingivalis*-related systemic comorbidities like cardiovascular disease (Chistiakov et al., 2016), pancreatic cancer (Fan et al., 2018), and Alzheimer’s disease (Dominy et al., 2019).

More than seventy years have passed since Joseph W. Bigger first named “persisters” in 1944 (Bigger, 1944). To date, persisters have been well-documented in nearly all bacterial species tested (Van den Bergh et al., 2017). Several clinical studies have proven their link to recalcitrant infectious diseases/conditions such as cystic fibrosis (Mulcahy et al., 2010), oral carriage (Lafleur et al., 2010), urinary tract infections (Goneau et al., 2014), and tuberculosis (Jain et al., 2016). Moreover, persisters create a suitable environment for gene transfer and adaptive mutation. For instance, *S. Typhimurium* persisters can act as a reservoir to promote resistance plasmid transfer among different microorganisms (Bakkeren et al., 2019), and the progeny of *E. coli* persisters harbor more antibiotic-resistant mutants (Barrett et al., 2019). Furthermore, intracellular bacterial persisters such as *Salmonella* could manipulate host immune response (Stapels et al., 2018). Due to these annoying roles persisters play in disease onset and development, how to eradicate persisters effectively has drawn a substantial amount of attention over the past two decades. It is known that developing new antibiotics has become rather tough since the 1980s, owing to high costs, being time-consuming, and great uncertainties of the outcomes. So, utilizing different strategies, like potentiating efficiency of conventional antibiotics and re-purposing the usage of classical antimicrobials, could be novel and realizable approaches (Allison et al., 2011; Barraud et al., 2013; Chung and Ko, 2019; Zhao et al., 2020).

Bismuth drugs such as colloidal bismuth subcitrate (CBS) are commonly used for treating *Helicobacter pylori* infection and related gastrointestinal disorders (Laine et al., 2003; Megraud, 2012; Dore et al., 2016). Other plausible applications of bismuth drugs have been increasingly reported, e.g., inhibiting metallo-β-lactamases-positive bacteria (Wang et al., 2018) and, very recently, suppressing SARS-CoV-2 replication (Yuan et al., 2020). The underlying action mechanisms of bismuth drugs have been increasingly understood in the past few years through adopting newly developed approaches such as metallomics and metalloproteomics. Bismuth binds to the key enzymes in the pathogens and subsequently disrupts the essential pathological pathways (Li et al., 2019; Griffith et al., 2021). Moreover, since bismuth acts as a broad-spectrum inhibitor of metallo-β-lactamases (MBLs), the combination of bismuth drugs with clinically used antibiotics could be an economical and effective alternative to fight against those antibiotic resistant mutations (Wang et al., 2018). Furthermore, bismuth drugs like CBS are frequently employed clinically, and the safety and toxicity issues have been well illustrated, showing that they only exert selective toxicity in microbes but not in human host (Li et al., 2019). We have newly demonstrated the potential effects of bismuth drugs on suppressing *P. gingivalis* in its various modes (Cheng et al., 2019), while whether these drugs can affect *P. gingivalis* persisters remains unknown and further investigation is highly warranted.

This study investigated the synergistic effects of a commonly used bismuth drug (CBS) combined with traditional antibiotics on the eradication of *P. gingivalis* persisters. Of note, *P. gingivalis* persisters were effectively eradicated in planktonic mode and nearly eliminated in biofilm mode, by CBS plus metronidazole. Whereas no such significant effects were observed for the combined usage of metronidazole and amoxicillin, or CBS and amoxicillin. Importantly, CBS exhibited no effects on the viability of HGECs, along with minimal cytotoxicity (<5%) even at a high concentration (400 μM). To the best of our knowledge, this is the first study revealing that *P. gingivalis* persisters could be well eliminated via the synergistic combination of a bismuth drug and metronidazole, thereby inspiring us to develop novel strategies and approaches to better control periodontitis and *P. gingivalis-*related systemic comorbidities.

## Materials and Methods

### Bacterial Culture

*P. gingivalis* (ATCC 33277) was cultured as previously described by us (Wang et al., 2020). *P. gingivalis* cells maintained as frozen stock were firstly grown on blood agar plates (44 g/L Columbia agar base, Difco, 5% horse blood, Hemostat, 5 mg/L hemin, Sigma-Aldrich, 1 mg/L vitamin K1, Sigma-Aldrich) in an anaerobic atmosphere composed of 10% H_2_, 5% CO_2_, and 85% N_2_ at 37°C. After 7-day culture, a single colony was picked into liquid trypticase soy broth (30 g/L TSB; Difco) supplemented with yeast extract (5 g/L), vitamin K1 (1 mg/L), and hemin (5 mg/L), and it was then cultured in the same anaerobic conditions.

### Antimicrobial Susceptibility

Antimicrobial susceptibility tests of *P. gingivalis* to metronidazole (MTZ, Sigma-Aldrich), Amoxicillin (AMX, Sigma-Aldrich), and colloidal bismuth subcitrate (CBS, De-Nol^®^) were performed as previously described following the CLSI guidelines (Clinical and Laboratory Standards Institute, 2016; Cheng et al., 2019). In brief, serial 2-fold dilutions of MTZ (0–50 mg/L), AMX (0–25 mg/L), or CBS (0–100 mM) were made for *P. gingivalis* culture broth in 96-well-plate (Thermo Fisher Scientific) with 50 μl in each well. Bacterial suspension (OD600 = 0.1) was added to each well (50 μl). The plates were incubated anaerobically at 37°C for 48 h. Herein, the minimal inhibitory concentration (MIC) was defined as the lowest concentration of antibiotic with no visible growth of *P. gingivalis*.

### Persister Assay

Persister assay was performed following our previous protocol with minor modifications (Li et al., 2018). *P. gingivalis* suspension was diluted in fresh media to OD 600 of 0.1 and incubated to stationary phase (72 h) followed by treatment with MTZ (100 mg/L), AMX (100 mg/L), CBS (100 μM), or different combinations of these medications. At 6, 24, and 48 h, the cultures were washed twice with PBS, followed by 10-fold dilution to 10^–7^ and 50 μl aliquots of each dilution were plated on blood agar plates for counting colony forming unit (CFU). The survival rate of persisters was then calculated via dividing the CFU of a drug-treated group by the untreated control group. Meanwhile, 3 μl aliquots of each dilution were spotted onto blood agar plates and anaerobically incubated for 7 days until further observation, and recorded.

The heritability of *P. gingivalis* persister was performed following an established approach (Li et al., 2018). *P. gingivalis* persisters after 24-h drug treatment were plated on blood agar plates to count CFU. A single colony was inoculated into fresh broth and cultured for 48 h. Then, the bacterial suspension was diluted in fresh media to OD600 of 0.1 and incubated to stationary phase (72 h) followed by treatment with the same drug. This process was repeated three times. The MIC of *P. gingivalis* recovered from the third treatment against AMX, MTZ, and CBS was determined as aforementioned.

### Drug Concentration Measurements

Drug concentration measurements were conducted with modified MIC assay. *P. gingivalis* in stationary phase (72 h) was treated with MTZ (100 mg/L), AMX (100 mg/L), and CBS (100 μM) for 48 h, respectively. After centrifugation (8,000 g, 10 min) and filtration (0.22 μm), the cultured broth was collected for testing. Herein, the broth was processed with serial 2-fold dilution (10 times) into 96-well-plate (50 μl/well), and *P. gingivalis* suspension (OD600 = 0.1) was added to each well (50 μl). The plates were incubated anaerobically at 37°C for 48 h. The drug concentration was measured according to the MIC results.

### Biofilm Assay

*P. gingivalis* in mid-exponential phase was diluted (OD600 of 0.1) and seeded onto Thermanoxt plastic coverslips (15 mm diameter, Thermo Fisher Scientific) on the bottom of 12-well plate.

The biofilms were firstly cultured for 72 h to reach maturation, followed by removing the free bacteria and treatment with MTZ (100 mg/L), AMX (100 mg/L), CBS (100 μM), or different combinations of these medications for 24, 48, and 72 h, respectively. After washing with PBS for three times, the biofilms were detected by plate culture. In brief, each plate with biofilm was put into 5 ml fresh broth and vigorously vortexed for 1 min. Then, the broth was 10-fold diluted with fresh broth to 10^–7^ and 50 μl aliquots of each dilution were plated on blood agar plates for counting CFU. Meanwhile, 3 μl aliquots of each dilution were spotted onto blood agar plates and anaerobically incubated for 7 days until further observation, and recorded.

### Cell Viability and Cytotoxicity Test

HGECs (CELLnTEC, Bern, Switzerland) were cultured and seeded into 96-well-plate (5 × 10^3^ cells/well) following our established protocol (Wang et al., 2020). After adhesion, cells were treated with different concentrations of CBS (0–400 μM) for 24, 48, and 72 h. Cell viability was evaluated with CyQUANT^TM^ MTT Cell Proliferation Assay Kit (Thermo Fisher Scientific Inc., United States), and the cytotoxicity was measured via Pierce CyQUANT^TM^ LDH Cytotoxicity Assay Kit (Thermo Fisher Scientific Inc., United States) concurrently according to the instructions of the products.

### Statistical Analysis

All experiments were undertaken for at least three independent repeats. Data were presented as the mean ± standard deviation (SD) for the results of independent experiments. GraphPad Prism 8 was used to take statistical calculations and obtain statistical graphs. Inter-group difference was compared using the One-Way or Two-way Analysis of Variance (ANOVA) and multiple comparisons by Tukey’s test as appropriate. Statistical significance was considered when *p* < 0.05.

## Results

### Multiple Antimicrobial Tolerance of *P. gingivalis* Persisters

**FIGURE 1:**
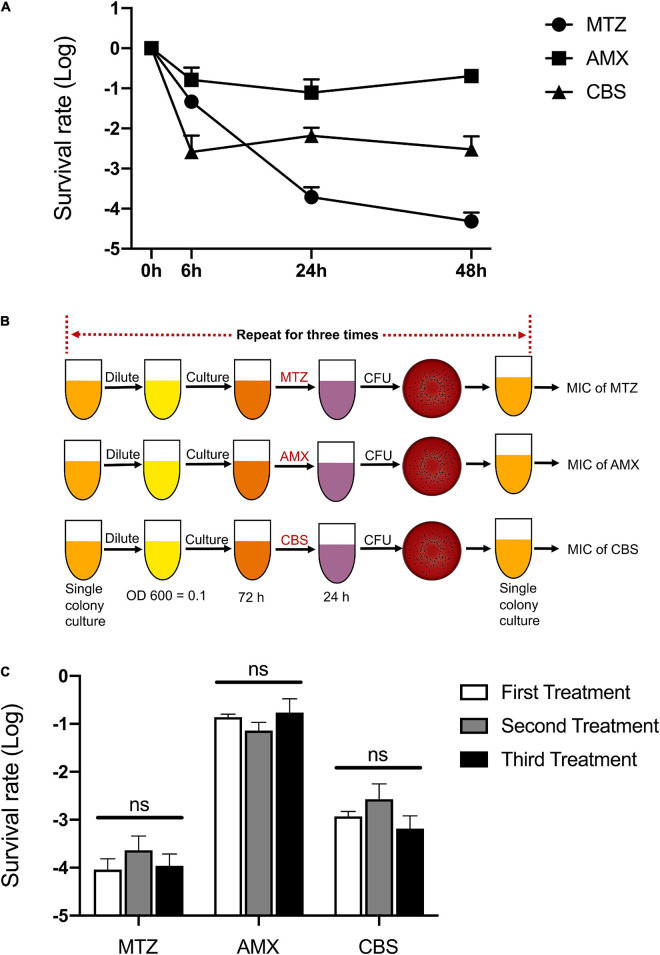
Multiple antimicrobial tolerance of *P. gingivalis* persisters. **(A)** The biphasic killing curves of *P. gingivalis* persisters after treatments with metronidazole (MTZ, 100 mg/L), Amoxicillin (AMX, 100 mg/L), and colloidal bismuth subcitrate (CBS, 100 μM) for 48 h, respectively. **(B)** Workflow of the three repeated treatments by MTZ, AMX, and CBS. **(C)** The survival rate of *P. gingivalis* persisters following the three repeated treatments by MTZ, AMX, and CBS. Data present the mean ± standard deviation (SD) of the results from three independent experiments. ns: no significance.

There was a biphasic pattern of killing curves against *P. gingivalis*, and a small subset of survival persisters existed after treatments with metronidazole (MTZ, 100 mg/L), Amoxicillin (AMX, 100 mg/L), and colloidal bismuth subcitrate (CBS, 100 μM) for 48 h (Figure 1A). In addition, these drugs remained effective against *P. gingivalis* during the experimental period (Table 1).

**TABLE 1:** Antimicrobial concentrations of MTZ, AMX, and CBS after 48-h treatment of *P. gingivalis.*

	0 h	48 h
MTZ (mg/L)	100 (256 × MIC)	6.25 (16 × MIC)
AMX (mg/L)	100 (512 × MIC)	25 (128 × MIC)
CBS (μM)	100 (16 × MIC)	25 (4 × MIC)

*MTZ, metronidazole; AMX, amoxicillin; CBS, colloidal bismuth subcitrate.*

In order to verify whether the surviving species contain resistant mutations, the treatments were repeated three times and the survival rates were calculated following every 24-h treatment. The MIC value of each antimicrobial was then tested after the last testing (Figure 1B). Notably, both survival rate and MIC value of *P. gingivalis* persisters remained unchanged (Figure 1C and Table 2).

**TABLE 2:** MICs of MTZ, AMX, and CBS against *P. gingivalis* persisters following 3-time treatments and recoveries^#^.

	Before	After
MTZ (mg/L)	0.39	0.39
AMX (mg/L)	0.20	0.20
CBS (μM)	6.25	6.25

*^#^Referring to Figure 1B.*

*MTZ, metronidazole; AMX, amoxicillin; CBS, colloidal bismuth subcitrate.*

### Colloidal Bismuth Subcitrate Plus Metronidazole Eradicated *P. gingivalis* Persisters in Planktonic Mode

**FIGURE 2:**
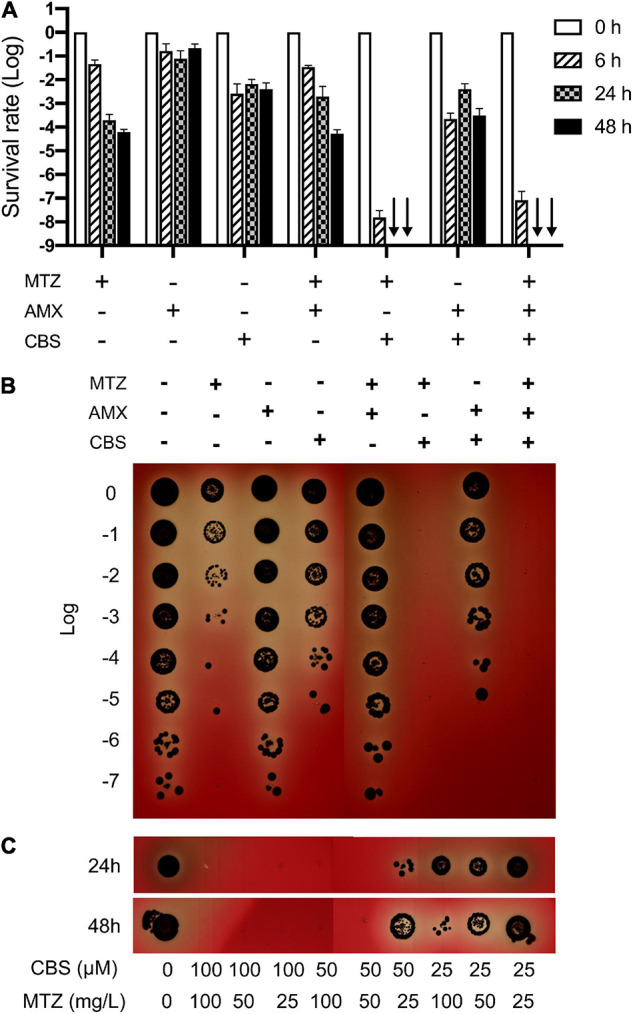
CBS plus MTZ eradicated *P. gingivalis* persisters in planktonic mode. **(A)** The survival rate of *P. gingivalis* persisters after treatments with MTZ (100 mg/L), AMX (100 mg/L), and CBS (100 μM) or different combinations of these medications for 6, 24, and 48 h, respectively. Data represent the mean ± SD of three independent experiments. Black arrows: not detected. **(B)** Plate culture of *P. gingivalis* persisters after 24-h treatment. **(C)** Combined usage of CBS and MTZ against *P. gingivalis* persisters with different concentrations of the drugs. **(B,C)** Show one randomly chosen result from three independent experiments.

No colony formation of *P. gingivalis* was observed after 24-h treatment of CBS plus MTZ, strongly indicating that its persisters were effectively eradicated through this combined approach. Indeed, it was much stronger than the traditionally used MTZ plus AMX or CBS plus AMX, as the persister cells survived even after 48-h treatments by these two sets of drugs (Figures 2A,B). Next, we further explored whether the combined usage of MTZ and CBS could reduce the anti-persister concentration of these drugs. Interestingly, it was highly achievable to get rid of the *P. gingivalis* persisters at relatively low dosages (25 mg/L of MTZ + 100 μM CBS; 50 mg/L of MTZ + 50 μM CBS) (Figure 2C).

### Colloidal Bismuth Subcitrate Plus Metronidazole Dramatically Suppressed *P. gingivalis* Persisters in Biofilm Mode

**FIGURE 3:**
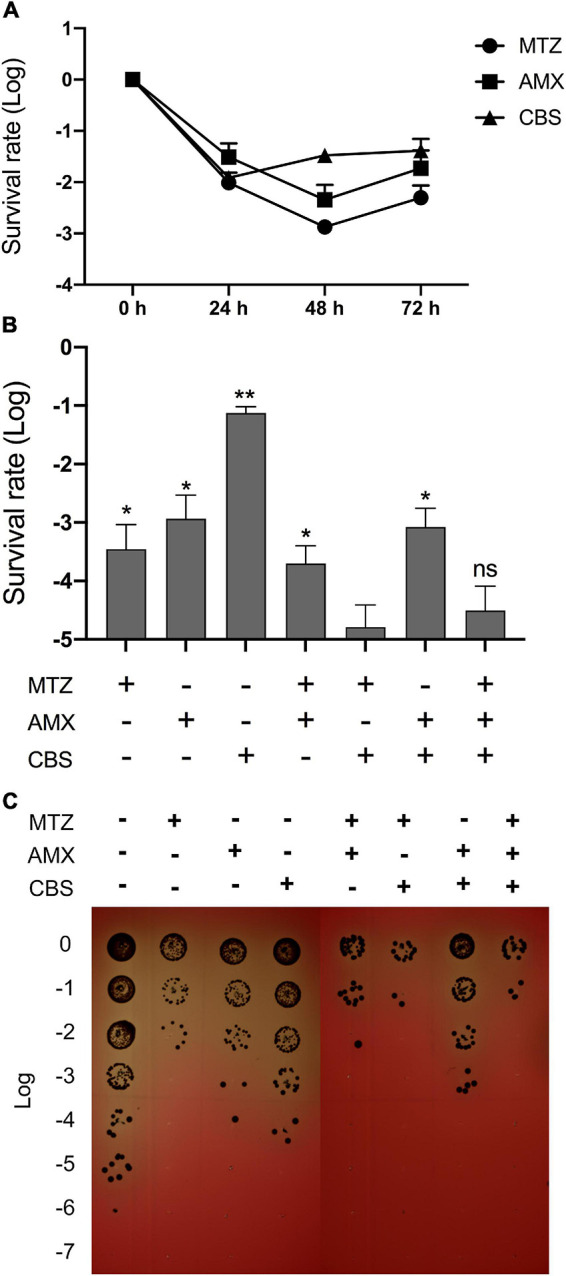
CBS plus MTZ dramatically suppressed *P. gingivalis* persisters in biofilm mode. *P. gingivalis* cells in biofilm mode were treated with MTZ (100 mg/L), AMX (100 mg/L), and CBS (100 μM) or different combinations of these medications for 24, 48, and 72 h, respectively. **(A)** The survival rate of *P. gingivalis* persisters after single drug treatment. **(B)** The survival rate of *P. gingivalis* persisters after 72-h treatment, with reference to the most effective group (MTZ + CBS). *P < 0.05; **P < 0.01; ns: no significance. Data in **(A,B)** represent the mean ± SD of three independent experiments. **(C)** Plate culture of *P. gingivalis* persisters after 72-h treatment, randomly chosen from three independent experiments.

It was found that single use of MTZ, AMX, or CBS was unable to eradicate *P. gingivalis* persisters in biofilm mode even after 72-h treatment (Figure 3A). Whereas these persisters were dramatically suppressed by the combined treatment of MTZ and CBS, thereby leaving a lower survival rate as compared to MTZ plus AMX or CBS plus AMX (Figures 3B,C).

### Colloidal Bismuth Subcitrate Exhibited Minimal Effects on Human Gingival Epithelial Cells

**FIGURE 4:**
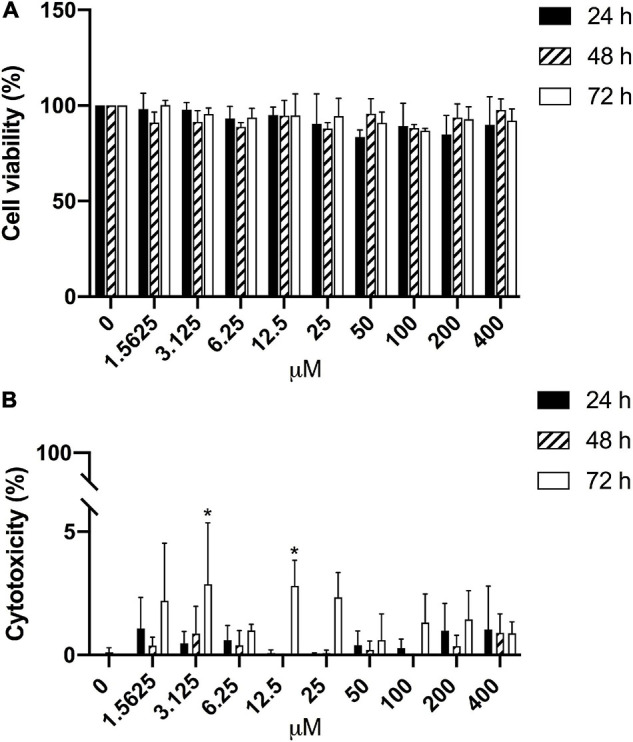
Negligible effects of CBS on HGECs. HGECs were treated with different concentrations of CBS for 24, 48, and 72 h, respectively. Cell viability and cytotoxicity were analyzed, with reference to the untreated group (0 μM). **(A)** Cell viability results from MTT test. No significant difference was found among different groups. **(B)** Cell cytotoxicity results from LDH test. *P < 0.05. Data represent the mean ± SD of three independent experiments.

Furthermore, the potential effects of CBS on HGECs were tested with different concentrations of CBS for 24, 48, and 72 h. Of note, the growth of HGECs was not affected (Figure 4A) and there was negligible cellular cytotoxicity (Figure 4B).

## Discussion

The initial study on “persister” dates back to the 1940s, while highly focused and intensive investigations on these noxious survivors started from the beginning of the twenty-first century (Lewis, 2001). Meanwhile, the widely used indwelling devices and increased numbers of immuno-compromised patients resulted in the explosion of chronic infections and inflammation, partly due to the action of various microbial persisters and current lack of relevant combating approaches (Lewis, 2010; Fauvart et al., 2011). It still remains a considerable challenge to effectively and predictably tackle persisters and related diseases (Putrins et al., 2015). Moreover, persisters also critically account for the emerging antimicrobial resistance worldwide (Barrett et al., 2019). It is therefore an urgent matter to seek novel strategies and approaches to eradicating persisters powerfully and precisely.

The past two decades have witnessed a booming increase in scientific research in the field of microbial persisters. Various novel agents have been developed against these tough persister cells. For instance, NH125, a well-known inhibitor of WalK, enables the successful removal of methicillin resistant *Staphylococcus aureus* persisters through inducing rapid membrane permeabilization (Kim et al., 2016). One unresolved issue is that plenty of time and money need to be invested for developing a new drug. Metallodrugs have been increasingly employed in medical healthcare. Of them, bismuth drugs have been routinely applied for managing patients with *Helicobacter pylori*-induced diseases (Marshall et al., 1988; Laine et al., 2003). Of note, our group has recently provided the first evidence that bismuth drugs can markedly suppress *P. gingivalis* in its planktonic, biofilm, and intracellular states (Cheng et al., 2019).

This study extended to investigate the potential effects of bismuth drug (CBS) on *P. gingivalis* persisters. Firstly, a single usage of MTZ, AMX, or CBS left a subset of survival persisters but not resistant mutations, and this outcome was not due to drugs failure. Importantly, the synergistic combination of CBS and MTZ completely eradicated the persister cells of *P. gingivalis*, while the MTZ plus AMX and CBS plus AMX could not. Furthermore, reduced dosages of both CBS and MTZ remained to totally eradicate *P. gingivalis* persisters. Thus, CBS could be an excellent alternative to potentiate the efficiency of MTZ for tackling *P. gingivalis* persisters.

In fact, a majority of the microbes are able to form biofilms, and therefore biofilm-related infections and inflammation are highly difficult to control (Lewis, 2007). Various underlying mechanisms have been proposed for explaining the high tolerance of biofilms to antimicrobials (Paraje, 2011; Singh et al., 2016; Teirlinck et al., 2017; Subramani and Jayaprakashvel, 2019; Uruen et al., 2020). Whereas it has been verified that the presence of persister cells crucially accounts for it (Lewis, 2001; Spoering and Lewis, 2001). Herein, Lewis sets a notable model to explain the biofilm-related recalcitrance of chronic infections that the complex microenvironment with microbial biofilms contributes to the formation of persisters. Indeed, antimicrobials along with the immune system could eradicate all the microorganisms outside the biofilms but frequently fail to eliminate the persisters within the biofilms, and these tough persisters could regrow following the termination of antimicrobial treatments (Lewis, 2007, 2010). As such, tackling persisters in the biofilms is critical for effective control of biofilm-related infections and inflammatory diseases. *P. gingivalis*, as the keystone periodontopathogen, co-aggregates with other oral pathogens to form multi-species biofilms to enhance survival capacity and pathogenicity (Bostanci and Belibasakis, 2012). Currently, it is rather challenging to suppress *P. gingivalis* in its biofilm mode. Our group has proved that bismuth drugs inhibit the formation of *P. gingivalis* biofilms and meanwhile significantly disrupt the mature biofilms (Cheng et al., 2019), while it remains unclear whether *P. gingivalis* persisters in their biofilm state could be suppressed. In this study, we found that single usage of MTZ, AMX, and CBS on the matured *P. gingivalis* biofilm was rather disappointing. Our study highlighted that CBS plus MTZ remarkably suppressed *P. gingivalis* persisters in their biofilm mode, with reference to MTZ plus AMX or CBS plus AMX. Further study is needed to refine the protocol for maximizing the anti-persister effectiveness in oral biofilms.

It is known that the serum concentration of bismuth after intake of one tablet of CBS can only reach a rather low level (44.5 μg/L or 0.2129 μM) (Vanhoe et al., 1993). In addition, the standard use of bismuth subcitrate, metronidazole, and tetracycline (BMT) for targeting *Helicobacter pylori* generates a relatively low concentration of blood bismuth (16.9 μg/L or 0.081 μM) (Guiard et al., 2019). Taken together, these findings indicate that such oral dosage of bismuth is far away from providing effective control of *P. gingivalis* in terms of its MIC (1,306 μg/L or 6.25 μM). Nevertheless, higher bismuth concentration in the serum would cause severe side-effects, such as encephalopathy, nephropathy, and osteoarthropathy (Slikkerveer and de Wolff, 1989; Cengiz et al., 2005). Thus, an appropriate approach is to deliver the bismuth drugs for topical application, such as through mouthwash, dentifrice, applying gels, or direct delivery to periodontal lesions, to enhance treatment efficiency while reducing side-effects. Our findings suggest CBS may be promising for treating periodontal diseases via local delivery mode, owing to its minimal effects on host cells like HGECs and negligible cellular cytotoxicity. Further translational studies should investigate the feasibility of combined usage of bismuth drugs with commonly used antibiotics for oral/periodontal healthcare in clinical practice.

It is apparent that metronidazole forms nitro radicals and generates toxic metabolites when entering into anaerobes, subsequently disrupting the DNA of microbial cells (Hernandez Ceruelos et al., 2019; Weir and Le, 2021). Anaerobes, meanwhile, could develop various intrinsic strategies to contract the drug action, such as overexpressing antioxidant enzymes like thioredoxin and SOD (Wassmann et al., 1999; Leitsch et al., 2016). Indeed, our previous work has proved that bismuth can continuously inhibit the activities of thioredoxin and SOD (Cheng et al., 2019). Thus, we suppose that the effective eradication of *P. gingivalis* persisters by CBS plus metronidazole could be due to their synergistic action on the oxidation-reduction reaction. Moreover, such a synergistic combination might be applicable to other anaerobic pathogens for tackling common chronic infections and inflammation. On the other hand, novel approaches need to be developed for refining the drug vehicles and maximizing the effectiveness. Our group has recently explored nano-based antimicrobials and anti-inflammatory agents, such as nanoparticle-encapsulated chlorhexidine and nanoparticle-encapsulated baicalein (Li et al., 2016, 2017). These findings are inspiring for further development along this line.

## Conclusion

This study indicates the existence of multi-drug tolerant *P. gingivalis* persisters, which is not due to antimicrobial resistance and drug failure. Notably, a synergistic combination of CBS and metronidazole sufficiently eliminates *P. gingivalis* persisters in planktonic mode, and remarkably suppresses their survival rates in biofilm mode. This combination is more effective than the commonly used metronidazole plus amoxicillin, or CBS plus amoxicillin. Importantly, CBS has minimal cytotoxic effects on HGECs and their viability is not affected. Our findings demonstrate that the synergistic combination of CBS and metronidazole enables the effective eradication of *P. gingivalis* persisters. This work may contribute to developing novel approaches to tackling *P. gingivalis* for effective control of periodontitis and common inflammatory comorbidities. Further investigation can be extended to tackle other pathogens for better care of common immuno-inflammatory diseases.

## Data Availability Statement

The original contributions presented in the study are included in the article/supplementary material, further inquiries can be directed to the corresponding author/s.

## Author Contributions

LJ conceived the project and revised the manuscript. CW and LJ designed the study. CW, XL, and TC performed the experiments, collected, and analyzed the data. CW drafted the manuscript. HS and LJ made a critical review of the manuscript. All authors contributed to the interpretation of the results and approved the final version of the manuscript.

## Conflict of Interest

The authors declare that the research was conducted in the absence of any commercial or financial relationships that could be construed as a potential conflict of interest.

## Publisher’s Note

All claims expressed in this article are solely those of the authors and do not necessarily represent those of their affiliated organizations, or those of the publisher, the editors and the reviewers. Any product that may be evaluated in this article, or claim that may be made by its manufacturer, is not guaranteed or endorsed by the publisher.
